# Estrategia para mejorar el acceso al tratamiento etiológico para la enfermedad de Chagas en el primer nivel de atención en Argentina

**DOI:** 10.26633/RPSP.2017.20

**Published:** 2017-04-13

**Authors:** Karen Klein, María Soledad Burrone, Juan Pedro Alonso, Lucila Rey Ares, Sebastián García Martí, Antonia Lavenia, Estela Calderón, Cynthia Spillmann, y Sergio Sosa Estani

**Affiliations:** 1 Instituto de Efectividad Clínica y Sanitaria (IECS) Instituto de Efectividad Clínica y Sanitaria (IECS) Buenos Aires Argentina Instituto de Efectividad Clínica y Sanitaria (IECS), Buenos Aires, Argentina.; 2 Programa Nacional de Chagas Programa Nacional de Chagas Argentina Programa Nacional de Chagas, Argentina.; 3 Instituto de Investigaciones Gino Germani Instituto de Investigaciones Gino Germani Buenos Aires Argentina Instituto de Investigaciones Gino Germani, Buenos Aires, Argentina.; 4 Programa Provincial de Chagas Programa Provincial de Chagas Tucumán Argentina Programa Provincial de Chagas, Tucumán, Argentina.; 5 Instituto Nacional de Parasitología “Dr. Mario Fatala Chabén” Administración Nacional de Laboratorios e Institutos de Salud, Ministerio de Salud. Consejo Nacional de Investigaciones Científicas y Técnicas Buenos Aires Argentina Instituto Nacional de Parasitología “Dr. Mario Fatala Chabén”, Administración Nacional de Laboratorios e Institutos de Salud, Ministerio de Salud. Consejo Nacional de Investigaciones Científicas y Técnicas, Buenos Aires, Argentina.

**Keywords:** Investigación, enfermedad de Chagas, tratamiento, descentralización, Argentina, Research, Chagas disease, therapy, decentralization, Argentina

## Abstract

**Objective:**

Improve distribution of etiological treatment of Chagas disease by identifying barriers to the decentralization of treatment to the first level of care in Argentina.

**Methods:**

A qualitative, exploratory, and descriptive study was conducted using semi-structured interviews of key actors belonging to the National Chagas Program and members of health teams at the first level of care, for the purpose of identifying barriers to diagnosis and treatment of Chagas disease at different levels (administrative, health agents, and community) that could affect a decentralized distribution strategy. Additionally, pilot decentralization was instituted in 10 primary health care centers in an Argentine province.

**Results:**

Semi-structured interviews were conducted with 22 program heads and health professionals. Principal obstacles found were lack of systematic case-finding, poor coordination among levels of care and health system actors, lack of health team training on treatment, patient monitoring, and patient-related barriers. A pilot decentralization program was carried out and strategies were evaluated to optimize large-scale intervention.

**Conclusions:**

The results made it possible to improve implementation of the plan to decentralize treatment through better inter-program coordination, capitalization on existing monitoring and communication tools, and sensitization of health teams. Furthermore, recommendations were developed to improve diagnosis and treatment of Chagas disease.

## RESUMEN

**Objetivo.:**

*Mejorar la distribución del tratamiento etiológico para la enfermedad de Chagas mediante la identificación de barreras para su descentralización al primer nivel de atención en Argentina*.

**Métodos.:**

*Se llevó a cabo un estudio cualitativo, de carácter exploratorio y descriptivo, en base a entrevistas semiestructuradas a actores clave pertenecientes al Programa Nacional de Chagas y miembros de los equipos de salud del primer nivel de atención con el objetivo de identificar barreras para el diagnóstico y tratamiento de la enfermedad de Chagas en diferentes niveles (administrativo, efectores de salud y comunidad) que podrían afectar una estrategia descentralizada de distribución. Además, se implementó un piloto de descentralización en diez centros de atención primaria en una provincia argentina*.

**Resultados.:**

*Se realizaron 22 entrevistas semiestructuradas con responsables de programas y profesionales de la salud. Los principales obstáculos hallados fueron la falta de búsqueda sistemática de casos, la poca articulación entre los niveles de atención y los actores del sistema de salud, la falta de capacitación del equipo de salud respecto al tratamiento, el seguimiento de los pacientes y las barreras asociadas a los pacientes. Se llevó a cabo un programa piloto de descentralización y se evaluaron estrategias para optimizar la intervención a gran escala*.

**Conclusiones.:**

*Los resultados permitieron mejorar la implementación del plan de descentralización del tratamiento a través de una mejor articulación interprogramática, la capitalización de herramientas de monitoreo y de comunicación ya existentes, y la sensibilización de los equipos de salud. Además, se formularon recomendaciones tendientes a mejorar el diagnóstico y el tratamiento de la enfermedad de Chagas*.

La enfermedad de Chagas es un problema grave de salud pública en América Latina, donde existe un total estimado de 6 millones de personas infectadas (1).

Las últimas estimaciones de casos en Argentina indican que habría aproximadamente 2 300 000 personas expuestas al Chagas, 1 500 000 infectadas (3,7% de la población) y más de 370 000 afectadas por cardiopatías de origen chagásico (1). El perfil epidemiológico se ha modificado en los últimos 15 años, con la transmisión congénita como la vía que genera mayor número de casos nuevos sobre la vectorial y la transfusional (2, 3). En 2014, la transmisión congénita sobre los niños estudiados y seguidos hasta el año de vida fue de 5,72% (4).

El objetivo del tratamiento etiológico es eliminar el parásito *(Trypanosoma cruzi)* de la persona infectada para disminuir la probabilidad de desarrollar manifestaciones clínicas de la enfermedad y romper la cadena de transmisión (5). La literatura demuestra que hay suficiente evidencia para la recomendación del tratamiento etiológico en el estadio agudo del Chagas (congénito o vectorial), como así también en cualquier estadio en menores de 19 años (5, 6).

El Programa Nacional de Chagas (PNCh) en Argentina (7) busca optimizar el diagnóstico temprano y el tratamiento oportuno de la infección aguda y crónica de Chagas, a través del diseño y distribución de guías de atención del paciente (8) y la compra y distribución gratuita de la medicación tripanocida desde el Ministerio de Salud de la Nación hacia los programas provinciales de Chagas. Estos, a su vez, los distribuyen a hospitales y centros de atención primaria de la salud (CAPS) —dependientes del subsistema público —según la demanda. El sistema de salud argentino cuenta con cobertura pública gratuita y universal, que coexiste con las obras sociales y sistema de medicina prepaga financiadas a través de esquemas de seguro obligatorios o voluntarios, respectivamente.

A pesar de la gratuidad y disponibilidad de la medicación, la prescripción médica para el tratamiento etiológico del Chagas ha sido históricamente baja, sobre todo en el primer nivel de atención (9, 10).

Por otra parte, no solo la prescripción de medicamentos ha sido baja en el primer nivel de atención, sino la aplicación de todas aquellas medidas vinculadas al control de la enfermedad. Para el tratamiento etiológico de la enfermedad de Chagas con tripanocidas, debe existir un control vectorial previo en la zona.

Por ello, el PNCh (11) y el Instituto Nacional de Parasitología (INP) (12), junto al Programa Remediar (que garantiza el acceso a medicamentos esenciales en el Sistema Público de Salud) (13), todos dependientes del Ministerio de Salud de la Nación, plantearon la necesidad de conocer las causas de esta problemática. Se pensó en una estrategia descentralizada de la distribución del tripanocida que mejore el acceso al tratamiento. En este contexto, se consideró oportuno desarrollar el presente estudio desde la perspectiva de la investigación en implementación para evaluar la mejor manera de llevar a cabo esta nueva estrategia.

El PNCh propone la descentralización de la medicación —que incluye la entrega del tripanocida y material sobre diagnóstico, tratamiento y farmacovigilancia —directamente a los CAPS a través del Programa Remediar, con el objetivo de incrementar el uso del medicamento y garantizar su reposición en forma adecuada. Esta estrategia implica no solo la distribución descentralizada, sino que esta sea apropiada a los requerimientos y necesidades de los CAPS, dado que se busca incrementar la tasa de prescripción en el primer nivel de atención.

Esta investigación se llevó a cabo durante el año 2015 y tuvo dos objetivos principales: el relevamiento de potenciales barreras para la descentralización del tripanocida mediante la realización de entrevistas a actores clave y la implementación de un programa piloto de descentralización en la provincia de Tucumán, Argentina.

**FIGURA 1.**
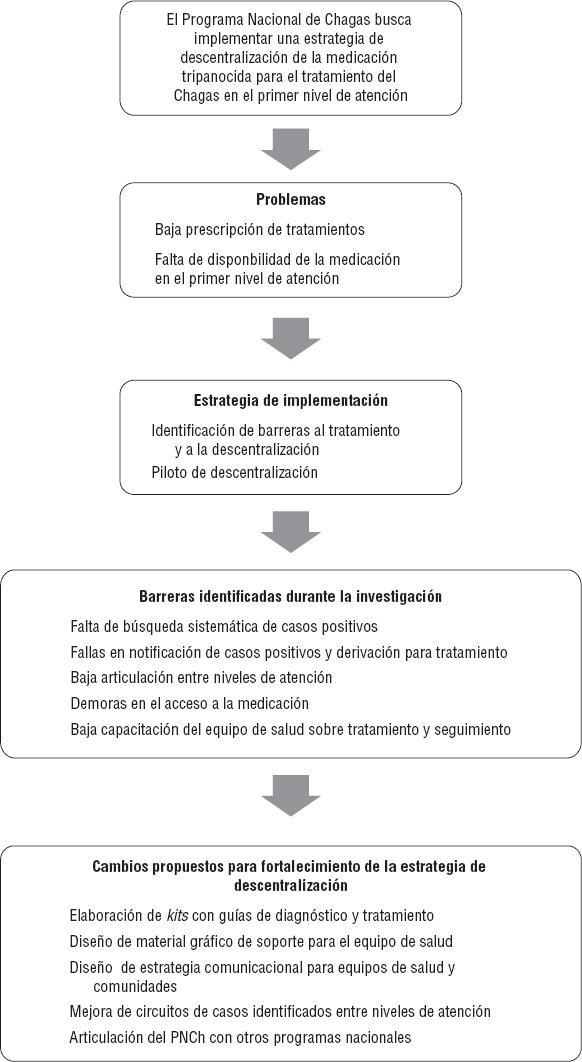
Fundamento para la propuesta de recomendaciones para fortalecer la estrategia de descentralización para el tratamiento de Chagas en Argentina

En la figura 1 se muestra de manera esquemática el problema que dio origen a este proyecto, así como las estrategias de implementación y cambios programáticos esperados. Los resultados obtenidos podrán ser utilizados para generar un plan de implementación de la descentralización a nivel nacional, con el fin de incrementar el uso de la medicación en la población objetivo.

PNCh, Programa Nacional de Chagas.

## MATERIALES Y MÉTODOS

### Diseño del estudio

El estudio forma parte de una nueva iniciativa llamada “Mejoras en la ejecución de programas a través de la investigación en implementación” (iPIER, del inglés *Improving Program Implementation through Embedded Research*), desarrollado por la Alianza para la Investigación en Políticas y Sistemas de Salud en colaboración con la Organización Panamericana de la Salud (OPS). Esta iniciativa coloca a los ejecutores de programas en el centro de una investigación con el objetivo de entender las fallas en los sistemas de salud que crean barreras a la implementación, lo que permite identificar soluciones y contribuye a la efectividad de los programas y políticas de salud (14). El presente trabajo se desarrolló con el diseño metodológico de investigación en implementación basada en dos abordajes. El primer abordaje está enfocado en la investigación, para el cual se utiliza una metodología cualitativa que identifique barreras de acceso al tratamiento etiológico de la enfermedad de Chagas en el primer nivel de atención. El segundo está enfocado en la implementación, para el cual se lleva a cabo el piloto de una intervención de descentralización en la provincia de Tucumán, endémica para Chagas.

El equipo de trabajo estuvo conformado por profesionales pertenecientes al PNCh e investigadores del Instituto de Efectividad Clínica y Sanitaria y del Instituto Gino Germani, ambos en Argentina.

El estudio fue aprobado por el Comité de Ética del Instituto Nacional de Parasitología y de la OPS. Los entrevistados consintieron su participación a través de la aceptación del consentimiento informado.

## Identificación de barreras

Para el relevamiento de las potenciales barreras se realizó un estudio cualitativo, de carácter exploratorio y descriptivo. Se realizaron entrevistas semiestructuradas a referentes nacionales y provinciales involucrados en el proceso de descentralización y a miembros de los equipos de salud del primer nivel de atención de distritos seleccionados. Se exploraron percepciones sobre las actuales barreras para el tratamiento del Chagas en el primer nivel de atención y sobre las potenciales barreras a la implementación de la descentralización.

Los informantes clave fueron seleccionados con la intención de incluir a los actores de los tres niveles de complejidad de atención, que se verán afectados por la descentralización. Los criterios de selección de los centros donde se realizaron las entrevistas fueron los mismos que se utilizaron para la selección de centros para el piloto. Para ello, se buscó representar heterogeneidad respecto a ubicación geográfica y si el personal recibió capacitación sobre el Chagas. A partir de estos, se seleccionaron intencionalmente cuatro centros: dos del interior y dos de la capital; uno de la capital y uno del interior recibieron capacitaciones, mientras los otros no. Los entrevistados fueron aquellos disponibles en el CAPS el día de la visita. Las entrevistas fueron grabadas y transcriptas literalmente. El texto fue analizado mediante técnicas de análisis cualitativo (análisis temático de contenido) ( 15) con el programa Atlas.ti^®^. Las entrevistas fueron codificadas a partir de los principales ejes y dimensiones abordados por el estudio, y el análisis temático estuvo orientado a identificar barreras a la implementación de la intervención.

## Piloto de descentralización

En lo referido a la implementación, se planificó la realización de un piloto en diez CAPS de una provincia endémica con la finalidad de evaluarlo y generar recomendaciones al PNCh para la implementación de un proceso efectivo de descentralización a nivel nacional. Para la inclusión de los CAPS se consideraron tres criterios. Uno fue la distribución según la jurisdicción y las áreas sanitarias (división de áreas programáticas con definición de población a cargo). Otro fue si los actores tuvieron o no capacitación sobre diagnóstico y tratamiento para Chagas. El tercero fue si habían brindado tratamiento en los cuatro años previos. Si bien el muestreo fue por conveniencia, se buscaron combinaciones diversas entre los tres criterios para tener una muestra heterogénea. Por último, el listado final de CAPS fue consensuado con referentes del PNCh, el INP y del Programa Provincial de Chagas. En febrero de 2016, se inició en estos diez CAPS la entrega descentralizada del tripanocida (que continúa hasta el presente), acompañada de material de comunicación diseñado por el PNCh para el equipo de salud y la comunidad (afiches, trípticos y las Pautas de diagnóstico y tratamiento del Chagas).

## RESULTADOS

Se realizaron 22 entrevistas semiestructuradas. Se entrevistó a seis actores clave en el diseño e implementación de la descentralización: responsables del PNCh y del INP, referentes de Programas Provinciales de Chagas y del Programa Remediar. Asimismo, se entrevistó a la coordinadora de un programa municipal de Chagas, referente de una asociación de personas infectadas del sur de la Provincia de Buenos Aires. Se entrevistó, además, a 15 miembros del equipo de salud, de diferentes especialidades y funciones (un director de CAPS, dos ginecólogos, dos pediatras, dos médicos generalistas, cinco agentes sanitarios, dos encargados de farmacia y un administrativo).

Las entrevistas permitieron identificar barreras para el diagnóstico y tratamiento del Chagas en el primer nivel de atención, en el sistema de salud, en los equipos de salud, y en los pacientes (cuadro 1). Se identificaron falencias en la notificación y los circuitos de atención de los casos positivos en el área programática. Los pacientes suelen ser diagnosticados en el segundo nivel de atención y no siempre reciben asesoramiento para continuar el tratamiento en centros de salud cercanos a sus domicilios. Esta deficiente articulación entre los niveles primario y secundario, y las fallas en el sistema de referencia y contrarreferencia, fueron señalados como aspectos problemáticos para el tratamiento y seguimiento de los pacientes en los CAPS.

**CUADRO 1.** Potenciales barreras para la efectividad de la intervención y recomendaciones para mejorar el diseño de implementación de la intervención en el tratamiento de la enfermedad de Chagas en Argentina

Barreras identificadas			Verbatims	Acciones a llevar a cabo
Administrativas o del sistema de salud	Poca articulación entre niveles de atención y actores del sistema desalud	Deficiencias en los circuitos de diagnóstico y tratamiento Comunicación deficiente entre niveles de atención y sectores Falencias en la notificación de casos positivos en el área programática	*Hay un montón de diagnosticados que efectivamente andan caminando por ahí, que no recibieron quizás orientación o indicación terapéutica cuando corresponde*. *Los pacientes se refieren al hospital (pero) no nos hacen contrarreferencia… tenemos que escarbar a ver qué es lo que está pasando con la paciente*.	Diseñar estrategias más fluidas y efectivas para mejorar el circuito entre los CAPS y los hospitales Fortalecer la articulación entre el Programa Nacional de Chagas y otros programas e instancias estatales dentro y fuera del sistema de salud, como el Programa Remediar, y el sector educativo, que tiene la obligación de realizar búsqueda activa de casos en zona endémica, de forma de consensuar acciones tendientes a mejorar la captación temprana y el tratamiento Mejorar la estrategia de registro y reporte de pacientes positivos (notificación y nominalización) para efectivizar el seguimiento de los mismos, a través del trabajo conjunto con el Sistema Nacional de Vigilancia en Salud (SNVS) y el Sistema Integrado de Información Sanitaria Argentina. Se plantea planificar un nuevo proyecto de investigación para evaluar una herramienta de notificación que permita un flujo de la información desde el PNA al SNVS y la generación de alertas hacia el PNA para el seguimiento del paciente
	Barreras en el acceso a la medicación	Falta de medicación en los CAPS Oportunidades perdidas de indicación de tratamientos.	Se demora el trámite de solicitar la medicación, por ahí al no tenerla directamente en el CAPS, hasta que llega pasa un tiempito, no mucho, pero bueno, hay que irlo a buscar al paciente…	Optimizar el monitoreo del funcionamiento de la descentralización a partir de la generación de preguntas específicas e indicadores adecuados para evaluar la estrategia
Equipo de salud	Barreras para el diagnóstico	Baja capacidad para diagnosticar nuevos casos en los CAPS. Poca visibilidad de la enfermedad de Chagas como problema en el equipo de salud Recursos limitados: turnos, laboratorio, entre otros	La verdad que no se buscan (casos) en forma sistemática, con análisis… No sé (si hay protocolos para tratamiento de Chagas), no te voy a decir porque no sé, yo nunca he leído, nunca en los últimos años…	Mejorar las capacitaciones que se efectúan desde el PNCh y los PPCh sobre el abordaje integral del Chagas Fortalecer la infraestructura vinculada a la atención del Chagas en el PNA
	Incertidumbre respecto a la indicación del tratamiento	Dudas sobre el responsable de indicar el tratamiento (especialista, generalista) Dudas sobre población objetivo	Hay una historia pasada de que el tratamiento no se impartía a todos, que ojo con el Chagas, que solo los agudos, después fuimos abriendo la cancha, pero siempre hay un grupo de gente que sigue con esta cuestión…	Clarificar a los prescriptores la población objetivo del tratamiento etiológico a través de las capacitaciones
	Deficiencias en la capacitación del equipo de salud en el tratamiento etiológico	Bajo conocimiento sobre indicación y seguimiento de tratamientos. Capacitación asistemática. Falta de capacitación de miembros no médicos del equipo de salud	*Van a tener que capacitar… yo no soy infectóloga, en mi vida he tratado un Chagas, siempre lo ha tratado el infectólogo*. Hemos ido a una capacitación hace mucho tiempo y no vamos todos, y eso es como que no nos sirve mucho, porque no podemos transmitir todo lo que hemos aprendido…	Mejorar las capacitaciones que se efectúan desde los PNCh y los PPCh sobre el abordaje integral del Chagas Ampliar la cobertura de las capacitaciones a enfermeros, promotores de salud, encargados de farmacia y otros profesionales del PNA
Pacientes	Barreras asociadas a los pacientes	Problemas de adherencia por efectos adversos Bajo conocimiento del Chagas	*La principal barrera es la adherencia del paciente… porque hay pacientes que dicen* “*me cayó mal, no la tomo más”, y ese es el drama*.	Diseñar una estrategia comunicacional dirigida a equipos de salud y comunidades con el fin de darle visibilidad a la problemática del Chagas y facilitar la indicación y el seguimiento del tratamiento Generación de materiales de divulgación de diferente tipo (folletería, afiches, aplicaciones de celular, entre otros) con información sencilla y concreta Modificar guías prácticas de prescripción de benznidazol (material nuevo y modificaciones de las guías de diagnóstico y tratamiento); por ejemplo, inclusión de tablas con la dosificación del fármaco según el peso corporal de los pacientes, o herramientas de fácil consulta para acudir en caso de dudas
Descentralización	Resistencias de responsables de PPCh a la descentralización	Temor a perder información Preocupación por la trazabilidad de la medicación Incertidumbre sobre el rol del PPCh	*Ahora es más fácil de controlar, de nominalizar la entrega, yo estoy sabiendo que la caja de medicación fue para estos pacientes, cuando estén en Remediar el gran desafío es ver qué pasa con el médico, que la medicación no quede en la vitrina de la farmacia*.	Fortalecer la articulación entre PNCh y los programas provinciales en el nuevo escenario de tratamiento descentralizado, explicitándose y consensuándose los roles y responsabilidades de cada una de las partes. Se espera que las contrapartes provinciales asuman una conducción más estratégica en la prevención del Chagas y la capacitación de los equipo de salud

CAPS, centros de atención primaria de la salud; PNCh, Programa Nacional de Chagas; PPCh, programas provinciales de Chagas; PNA, primer nivel de atención.

Se señalaron también falencias en el acceso a la medicación en el esquema centralizado, por demoras en el envío de la medicación y por oportunidades de tratamiento perdidas al no disponer de medicación en los efectores. También se identificaron dificultades en la búsqueda y el diagnóstico de casos nuevos. A excepción de tamizajes en mujeres embarazadas, no se realiza búsqueda activa en la población de adultos o niños. Aunque se trata de una región endémica, la enfermedad de Chagas no se cuenta entre las principales preocupaciones de los equipos de salud. Otro obstáculo mencionado fue la deficiente formación de los equipos de salud del primer nivel de atención en el tratamiento de la infección con *T*. *cruzi*. Varios profesionales refirieron una escasa experiencia clínica y bajo conocimiento para indicar y supervisar tratamientos, así como la falta de capacitación de miembros no médicos del equipo. También fue señalada como una potencial barrera la falta de transferencia de información y saberes entre los profesionales que asistieron a las capacitaciones y aquellos que no lo hicieron.

Como factores ligados a la comunidad, se mencionó el bajo conocimiento de la población sobre la enfermedad de Chagas y problemas de adherencia al tratamiento ligados a los efectos secundarios o al poco seguimiento del tratamiento por parte del equipo de salud.

La valoración de la estrategia de descentralización fue variable. Para algunos facilitaría *per se* el acceso de los pacientes al tratamiento, mientras que otros fueron más cautos respecto a las potenciales ventajas por sobre la distribución actual. Los entrevistados coincidieron en que esta debería ser acompañada por otras intervenciones, como mejorar esfuerzos diagnósticos, capacitar al equipo de salud (capacitaciones más sistemáticas dirigidas a todos sus miembros), distribuir material de apoyo con herramientas de fácil consulta y realizar tareas de divulgación en la comunidad. Entre las barreras a la implementación de la descentralización se identificaron resistencias e inquietudes de los responsables de los programas provinciales de Chagas, por posibles pérdidas de información y la incertidumbre sobre el rol de los programas locales ante el nuevo escenario. Para los responsables de programas consultados, los resultados del estudio BENEFIT (16), que mostró que el tratamiento tripanocida en pacientes con cardiopatía chagásica instalada no reduce de manera significativa el deterioro clínico, podría generar incertidumbre en los equipos de salud, pero no afectaría los objetivos de la descentralización, dado que esta busca reforzar el tratamiento en los casos en que ya existe evidencia de beneficio.

También se relevaron opiniones acerca de las estrategias de monitoreo de los tratamientos, a realizarse con formularios actualmente utilizados por el Programa Remediar para otros fármacos, donde se registran la prescripción y dispensa de la medicación. Si bien no proveen información sobre la finalización del tratamiento o sobre los efectos secundarios, varios entrevistados señalaron las ventajas comparativas con los registros actuales. La familiaridad de los equipos de salud con estas herramientas facilitaría la adopción del monitoreo.

La implementación del piloto incorporó aportes del estudio cualitativo e implicó la realización de cambios tanto al interior del PNCh como en su relación con otros programas y organismos (cuadro 2). En primer término, se fortaleció la articulación con el Programa Remediar: se realizó la selección y desarrollo de la herramienta de recolección de información de prescripción y uso del tripanocida a través de una modificación en la herramienta ya existente en el programa (13) para el control de uso y *stock* de medicación, que permite cuantificar la prescripción y fortalecer un mecanismo de reposición de la medicación en el primer nivel de atención. Además, se propuso al PNCh continuar con la evaluación de la implementación para medir su efectividad.

**CUADRO 2.** Principales cambios realizados a partir del estudio “Estrategia para mejorar acceso al tratamiento etiológico para Chagas en el primer nivel de atención en Argentina”

Nivel	Cambios
Nivel interprogramático	Fortalecimiento de la articulación entre programas de nivel nacional
	Articulación entre el programa provincial de Chagas y la sede del Programa Remediar en la provincia de Tucumán
	Fortalecimiento de la articulación con el equipo técnico del PNCh y el equipo de consultores del Proyecto de Fortalecimiento de la interrupción de la transmisión vectorial de la enfermedad de Chagas en Argentina, junto al equipo provincial de Chagas
Nivel programático	Capitalización de herramientas ya existentes para la recolección de información de prescripción y uso del tripanocida
	Implementación de un sistema de monitoreo de la estrategia de descentralización y prescripción
	Articulación con las áreas de información, educación y comunicación del PNCh para brindarle los resultados obtenidos en este estudio y diseñar una estrategia comunicacional dirigida a equipos de salud y comunidades con el fin de darle visibilidad a la problemática de Chagas y facilitar la indicación y el seguimiento del tratamiento
	Inclusión del benznidazol en una modalidad de distribución de medicación previamente existente en el Programa Remediar, que abarata costos de envío paralelos
	Desarrollo de indicadores adecuados para evaluar el proceso de descentralización
	Desarrollo de un sistema de monitoreo del funcionamiento de la descentralización a partir de la aplicación de los indicadores por el equipo técnico del PNCh
Nivel comunitario y del sistema de salud	Sensibilización a los diversos actores del primero, segundo y tercer niveles sobre la actual brecha en el tratamiento y la importancia de tratar a los pacientes con Chagas, según normas

PNCh, Programa Nacional de Chagas.

A partir de los resultados obtenidos en la identificación de barreras y con la puesta en marcha del piloto se realizaron recomendaciones para mejorar el diseño de la implementación de la intervención (cuadro 1 y figura 1).

## DISCUSIÓN

La investigación cualitativa evidenció barreras que dificultan el diagnóstico y el tratamiento etiológico de la enfermedad de Chagas en el primer nivel de atención.

La producción de investigación cualitativa respecto a esta temática es escasa (17, 18 ). Los estudios se concentran principalmente en la prevención y el control del vector, con el abordaje de las actitudes y el conocimiento de la población general, de personas infectadas y de grupos vulnerables ( 19-21).

En este estudio, se hallaron fallas en la búsqueda sistemática de nuevos casos, en la notificación y registro, así como en el tratamiento y seguimiento de los casos positivos. Manne-Goehler et al. (22) coinciden y atribuyen esto tanto a la falta de conocimiento y priorización del problema por parte de los médicos y la comunidad, como a la poca claridad en las recomendaciones de cuidado y a la escasez de recursos en los servicios. Respecto a las capacitaciones, en este trabajo se señalan, como muestran estudios sobre el tema (22, 23 ), debilidades en la formación de los equipos de salud respecto a la indicación y seguimiento de los tratamientos. Las actividades de capacitación deberían incluir a personal médico y no médico, como personal de enfermería y promotores de salud, que podrían cumplir un rol importante en la detección y acompañamiento de los casos positivos.

También sería necesario fortalecer el empoderamiento de la comunidad, dado que se mencionaron el bajo conocimiento y preocupación de la población sobre la enfermedad, además de problemas de adherencia al tratamiento ligados a los efectos secundarios. Explorar mejor estas barreras en futuros estudios es fundamental para lograr un tratamiento efectivo que permita reducir la carga de enfermedad.

Respecto a la claridad de las recomendaciones, tres estudios dan especial relevancia a la necesidad de contar con guías o recomendaciones claras ( 22-24). La historia de controversias sobre qué pacientes deberían ser tratados, en la que se inscribe la reciente actualización de las recomendaciones movilizada por el estudio BENEFIT (16), ha contribuido a generar incertidumbre respecto a la indicación del tratamiento. Dichas actualizaciones requieren una amplia difusión de los nuevos protocolos entre los proveedores de salud que deberían indicar los tratamientos.

Otra dificultad señalada para tratar y seguir a los infectados es la falta de notificación de casos (23), por lo que los registros y el reporte de casos al sistema de vigilancia en salud debería mejorarse (25, 26 ). También la falta del tripanocida en el efector de salud en el primer nivel de atención es una barrera de gran peso reconocida en estos estudios (22-24).

La escasa articulación entre los niveles de atención y las demoras en el acceso a la medicación fueron otras barreras señaladas por los entrevistados. Villa et al. (24) concluyen en su artículo que el cuidado del paciente debería centralizarse en el primer nivel de atención, con una participación articulada de los otros niveles. Priorizan también tener disponibilidad de la medicación en el servicio para mejorar las tasas de tratamiento, lo que apoyaría la estrategia de descentralización propuesta en este estudio.

Las opiniones sobre los potenciales obstáculos en el proceso de implementación de esta estrategia de descentralización fueron variadas. Algunos entrevistados consideraron que la intervención facilitaría el acceso al tratamiento, mientras que otros expresaron la preocupación de que la descentralización por sí sola no mejore la situación actual. Las inquietudes de los responsables de los programas provinciales de Chagas es un dato a tener en cuenta para anticipar resistencias a la estrategia de descentralización. Los participantes coincidieron en la necesidad de complementar la intervención con acciones destinadas a los prescriptores, al equipo de salud en su totalidad y a la comunidad (capacitación, disponibilidad de recursos de diagnóstico y seguimiento, herramientas de comunicación y divulgación en los CAPS y la comunidad). Asimismo, la utilización de los formularios del Programa Remediar representaría una contribución significativa en el monitoreo de los tratamientos.

Entre las limitaciones del estudio pueden señalarse los problemas administrativos para evaluar la prueba piloto en los tiempos previstos, actividad que se prevé completar con el apoyo del PNCh. Por otra parte, debido a las características de la investigación cualitativa, los resultados no pueden generalizarse a todos los centros de salud del país. Las barreras identificadas, sin embargo, permitieron definir con mayor precisión potenciales obstáculos en la implementación de la descentralización y delinear acciones tendientes a superarlos.

Los resultados del estudio y el proceso iniciado de la implementación del piloto generaron cambios concretos en el programa y sirvieron como insumos en el diseño de intervenciones puntuales que deberían acompañar la estrategia de descentralización para mejorar su efectividad. Se propuso al PNCh continuar con la evaluación del piloto para conocer su efectividad y, para ello, se articuló con el equipo técnico del Programa y el equipo de consultores del Proyecto de Fortalecimiento de la interrupción de la transmisión vectorial de la enfermedad de Chagas ( 27, 28 ).

Además, se inició el proceso de actualización de las pautas oficiales de atención del paciente infectado con *T*. *cruzi* en Argentina y se comenzó a actualizar el material gráfico de soporte para el equipo de salud vinculado a la indicación de tratamiento. Se articuló con las áreas de información, educación y comunicación del PNCh para diseñar una estrategia comunicacional dirigida a equipos de salud y comunidades con el fin visibilizar la problemática de Chagas y facilitar la indicación y seguimiento del tratamiento. Se recomendó y puso a consideración la generación de materiales de divulgación de diferente tipo (folletería, aplicaciones de celular, entre otros) con información sencilla y concreta para los equipos de salud y para los pacientes acerca de la enfermedad, el tratamiento y el seguimiento.

Asimismo, se propuso una serie de recomendaciones destinadas a diversos actores del sistema de salud, tendientes a mejorar las condiciones para el diagnóstico y el tratamiento de la enfermedad de Chagas. Las mismas fueron elevadas a los referentes del PNCh y se contemplaron estrategias para la continuidad de la evaluación de la intervención —una vez que esta pueda implementarse en su totalidad— y para el diseño de un plan nacional sustentable a largo plazo.

## CONCLUSIONES

El presente estudio permitió iniciar la implementación de una nueva estrategia de distribución de la medicación tripanocida y generar cambios a nivel programático y de los servicios de salud. También se identificaron barreras y se propusieron mejoras a la prescripción del tratamiento etiológico de la enfermedad de Chagas en el primer nivel de atención, y a la adopción y efectividad de la estrategia de descentralización, con vistas al escalamiento a nivel nacional.

Fortalecer la articulación interprogramática y entre los niveles de atención, mejorar las capacitaciones y la estrategia comunicacional para equipos de salud y comunidades, y poner a disposición el tripanocida en el centro de salud mediante la nueva estrategia de descentralización, así como facilitar el acceso a guías de diagnóstico, tratamiento y seguimiento claras, son medidas fundamentales para mejorar el acceso a cuidados apropiados de los pacientes con enfermedad de Chagas.

## Agradecimientos.

El equipo de trabajo del presente estudio agradece a los funcionarios del Instituto Nacional de Parasitología de Argentina, que incentivaron la iniciativa y posibilitaron llevar adelante esta investigación; a referentes del Programa Nacional de Chagas de Argentina y de los programas provinciales participantes. Asimismo agradecemos a Anabel Fernández, responsable de la Unidad de Seguimiento y Evaluación del Programa Remediar, a los profesionales de salud y a otros informantes clave que aceptaron participar en las entrevistas. Por último, el equipo agradece a Claudio Moreno, quien facilitó el transporte y garantizó el traslado para la realización del trabajo en terreno.

## Financiamiento del estudio.

El estudio “Estrategia para mejorar el acceso al tratamiento etiológico para Chagas en el primer nivel de atención en Argentina” fue financiado por la Organización Panamericana de la Salud.

## Declaración.

Las opiniones expresadas en este manuscrito son responsabilidad del autor y no reflejan necesariamente los criterios ni la política de la *RPSP/PAJPH* y/o de la OPS.
